# Estrogen-related mechanisms in sex differences of hypertension and target organ damage

**DOI:** 10.1186/s13293-020-00306-7

**Published:** 2020-06-01

**Authors:** Andrea Rodrigues Sabbatini, Georgios Kararigas

**Affiliations:** 1 Cell Signalling Research Group, School of Life Sciences, University of Nottingham, Nottingham, UK; 2 Charité – Universitätsmedizin Berlin, corporate member of Freie Universität Berlin, Humboldt-Universität zu Berlin, and Berlin Institute of Health, Berlin, Germany; 3 DZHK (German Centre for Cardiovascular Research), partner site Berlin, Berlin, Germany

**Keywords:** Blood pressure, Cardiovascular, Heart, Sex hormone, Vasculature

## Abstract

Hypertension (HTN) is a primary risk factor for cardiovascular (CV) events, target organ damage (TOD), premature death and disability worldwide. The pathophysiology of HTN is complex and influenced by many factors including biological sex. Studies show that the prevalence of HTN is higher among adults aged 60 and over, highlighting the increase of HTN after menopause in women. Estrogen (E2) plays an important role in the development of systemic HTN and TOD, exerting several modulatory effects. The influence of E2 leads to alterations in mechanisms regulating the sympathetic nervous system, renin-angiotensin-aldosterone system, body mass, oxidative stress, endothelial function and salt sensitivity; all associated with a crucial inflammatory state and influenced by genetic factors, ultimately resulting in cardiac, vascular and renal damage in HTN. In the present article, we discuss the role of E2 in mechanisms accounting for the development of HTN and TOD in a sex-specific manner. The identification of targets with therapeutic potential would contribute to the development of more efficient treatments according to individual needs.

## Background

Hypertension (HTN) is a multifactorial condition affecting around 1.13 billion people worldwide, with an estimated increase of 15–20% by 2025, reaching close to 1.5 billion [1, 2]. HTN is a primary risk factor for cardiovascular (CV) events, target organ damage (TOD), and premature death and disability worldwide [3–6]. Individual characteristics, such as age, race, body mass and genetic factors, as well as environmental factors, lifestyle and dietary habits, such as salt intake, may contribute to the development of HTN and TOD, i.e., cardiac, vascular and renal damage.

Notably, biological sex has been revealed as a key factor in understanding variation in the development of HTN and related CV implications. Recent data show that men have a higher prevalence of HTN than women among adults aged 18–39 years (9.2% men vs. 5.6% women) and 40–59 years (37.2% men vs. 29.4% women), but men have a lower prevalence of HTN than women in adults older than 60 years (58.5% men vs. 66.8% women) [7]. Therefore, aging is characterized by increases in blood pressure (BP) in both men and women, reaching 63.1% among adults aged 60 years and over, and it is well known that the incidence of HTN increases after menopause in women [8]. Actually, women experience steeper increases in BP than men as they age [9].

Along this line, 41% of postmenopausal women become hypertensive, while more than 75% of women older than 60 years are hypertensive in the USA [10]. The majority of women older than 60 years has stage 2 HTN (BP ≥ 160/100 mmHg) or receives antihypertensive therapy [11–13]. Notably, it is more difficult to achieve BP control in elderly women, and women are at a greater risk of developing resistance to antihypertensive treatment than men [3, 14]. Due to the dramatic increase of HTN in postmenopausal women, it is expected that the steroid hormone estrogen plays an important role in this process.

**Fig. 1:**
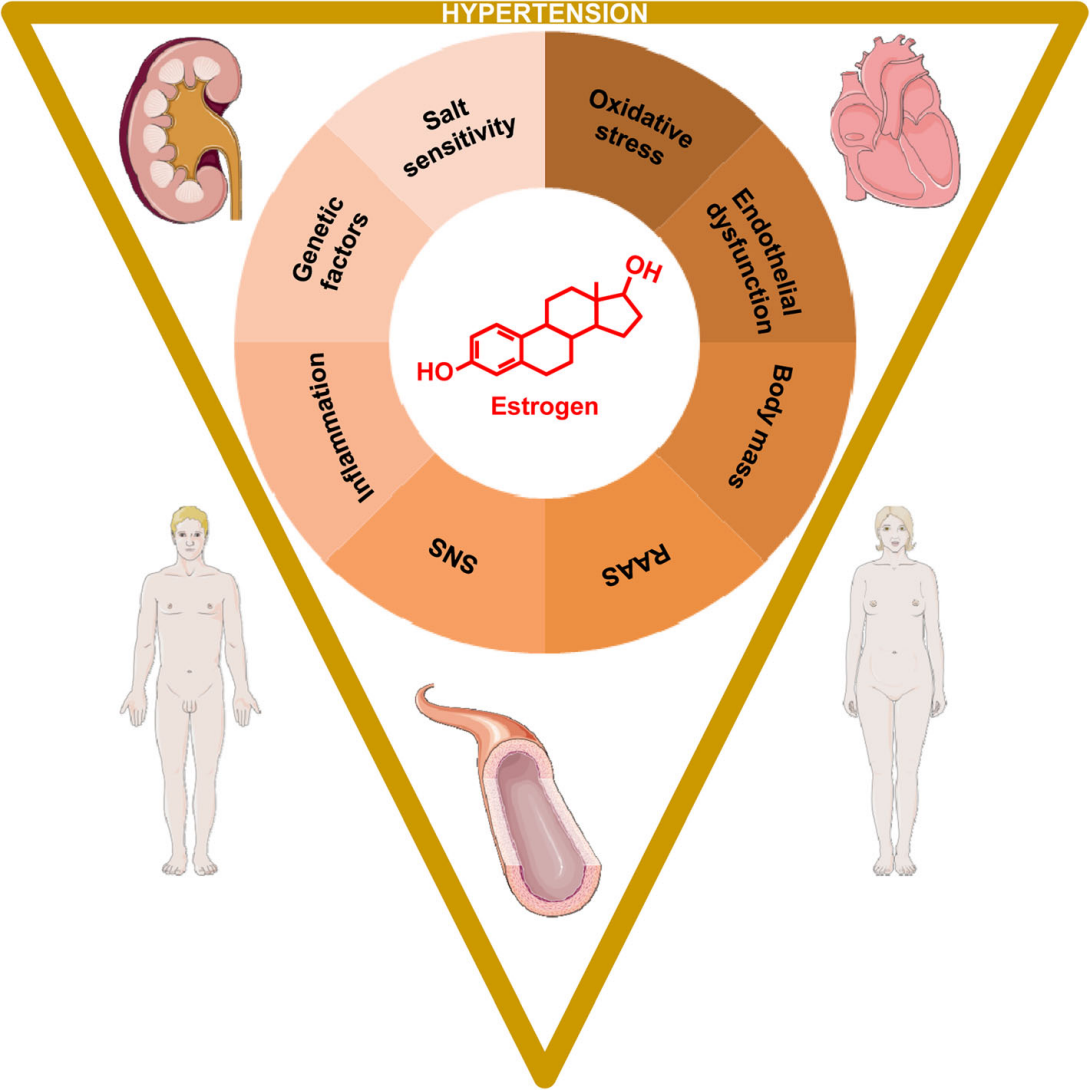
Role of estrogen in sex differences in hypertension and related target organ damage. The influence of estrogen leads to alterations in mechanisms regulating the sympathetic nervous system (SNS), renin-angiotensin-aldosterone system (RAAS), body mass, endothelial (dys) function, oxidative stress and salt sensitivity; all associated with a crucial inflammatory state and influenced by genetic factors, ultimately resulting in cardiac, vascular and renal damage in hypertension in a sex-specific manner

In fact, several studies have investigated the influence of 17β-estradiol (E2) in the development of systemic HTN and TOD. The influence of E2 leads to alterations in mechanisms regulating the sympathetic nervous system (SNS), renin-angiotensin-aldosterone system (RAAS), body mass, oxidative stress, endothelial function and salt sensitivity; all associated with a crucial inflammatory state and influenced by genetic factors, ultimately resulting in cardiac, vascular and renal damage in HTN. In the present article, we discuss the role of E2 in mechanisms accounting for the development of HTN and TOD in a sex-specific manner (Fig. 1). Accordingly, the goal of this article is not to provide an exhaustive review of the literature, but rather to focus on pertinent studies (Table 1).

**Table 1:** Examples of pertinent findings of estrogen administration

Effect	Sample	Reference
Increased baroreflex sensitivity	Postmenopausal women	[15]
Decreased renin levels & ACE activity	Postmenopausal women	[16, 17]
Increased eNOs & NO	Human umbilical vein & bovine aorta endothelial cells	[18]
Decreased sICAM1, VCAM1, IL-6 and plasma E-selectin	Postmenopausal women	[19]
Decreased left ventricular mass	Postmenopausal women	[20]
Decreased cardiomyocyte surface area	Neonatal rat cardiomyocytes	[21]
Accumulated nuclear phosphorylated protein kinase B	Neonatal rat cardiomyocytes	[22]
Decreased collagen; increased elastin	Primary human aortic smooth muscle cells	[23]
Increased leptin sensitivity; decreased insulin sensitivity	Ovariectomized Long-Evans rats	[24]
Decreased proteinuria, TGFB1 & PDGFA	Ovariectomized Wistar rats	[25]

*ACE* angiotensin-converting enzyme, *eNOs* endothelial NO synthase, *IL-6* interleukin-6, *NO* nitric oxide, *PDGFA* platelet-derived growth factor subunit A*, sICAM1* soluble intercellular adhesion molecule 1*, TGFB1* transforming growth factor beta 1*, VCAM1* vascular cell adhesion molecule 1

## Regulatory effects of estrogen in hypertension

### Sympathetic nervous system

**Fig. 2:**
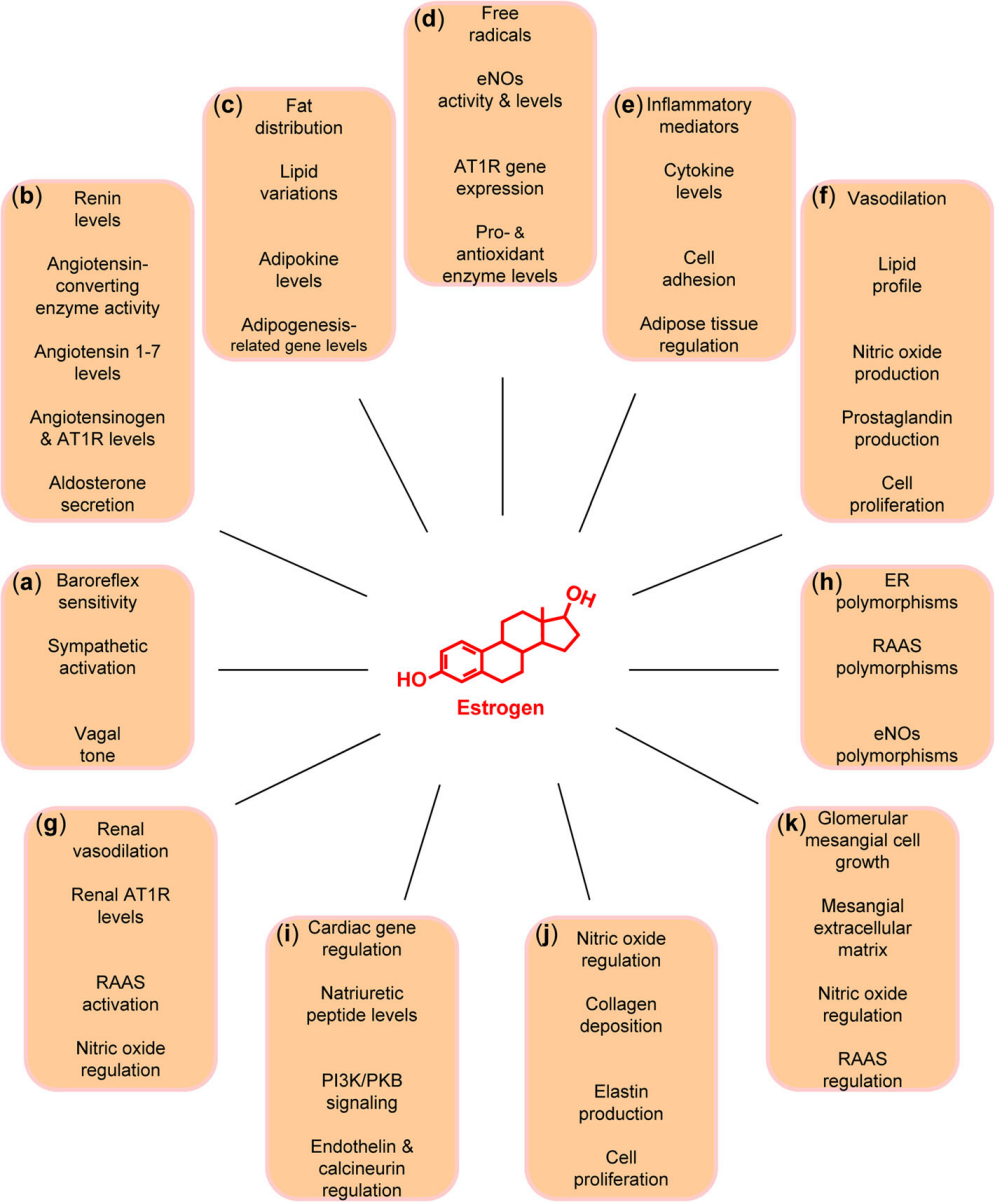
Regulatory effects of estrogen leading to sex differences in hypertension: **a** sympathetic nervous system, **b** renin-angiotensin-aldosterone system, **c** body mass, **d** oxidative stress, **e** inflammation, **f** endothelial (dys) function, **g** salt sensitivity, **h** genetic factors, **i** cardiac hypertrophy, **j** arterial stiffness, and **k** renal dysfunction. AT1R, angiotensin type 1 receptor; eNOs, endothelial nitric oxide synthase; ER, estrogen receptor; PI3K, phosphoinositide 3-kinase (PI3K); PKB, protein kinase B (also known as AKT); RAAS, renin-angiotensin-aldosterone system

The role of the SNS in the development of HTN is well established, mediated by renal sympathetic nerves, increased renin release, alteration of glomerular filtration rate, and increased tubular sodium reabsorption [26]. Sympathetic nerve activity decreases with age, but it increases in the presence of weight gain and metabolic syndrome [27, 28], common to postmenopausal women. In addition, sympathetic nerve activity differs significantly between men and women [29] and E2 is expected to mediate sex differences by exerting several regulatory effects (Fig. 2a).

In this context, E2 has an important role in the brain. Peripheral efferent nerves and signaling pathways that respond to neurotransmitters and neurons containing nuclear estrogen receptors (ER) have been identified in brain centers involved in the regulation of CV function [30–32]. Interestingly, lower baroreflex sensitivity has been described in postmenopausal women compared with age-matched men, while postmenopausal women with hormone therapy (HT) had higher baroreflex sensitivity than those not on HT [15]. Along this line, it was suggested that E2 exerts direct effects on central nervous autonomic centers, thereby leading to sympathoinhibitory effects, which could be important for conferring CV protection [33].

Several studies with experimental animals support this notion. For example, ovariectomized (OVX) rats showed enhanced sympathetic activation and attenuated baroreflex sensitivity or vagal tone, while these effects were attenuated with E2 treatment [34]. It was further shown that the activation of ERβ with a selective agonist in the paraventricular nucleus and rostroventrolateral medulla of OVX rats attenuates the sympathetic nerve activity reducing BP in aldosterone-induced HTN [35], suggesting that a potential decrease in ERβ levels or function with aging could contribute to SNS-mediated HTN in women. Furthermore, E2 treatment inhibited the development of left ventricular (LV) hypertrophy (LVH) in baroreceptor-denervated rats [36]. Together, these data highlight the regulatory role of E2 in the SNS and its influence in the development of CV pathology related to HTN.

The renal sympathetic nerves have also been shown to play a role in regulating HTN in young and old female spontaneously hypertensive rats (SHR), with a greater decrease in BP with adrenergic blockade occurring in old compared with young animals [37], suggesting an important contribution of the SNS to HTN in old animals. In addition, renal denervation was associated with reduced BP in both young and old females, with a more pronounced response in old females. However, after renal denervation the BP remained above 140 mmHg [37–39], indicating that mechanisms other than the renal nerves also contribute to HTN. In humans, clinical trials of renal denervation for resistant HTN showed inconsistent BP results [40–42]. However, a revised procedural method in the absence of antihypertensive treatment led to significant BP reductions [43]. Nevertheless, the efficacy of renal denervation in the setting of concurrent antihypertensive treatment was challenged [44, 45], but the latest findings showed a significant reduction in BP [46].

### Renin-angiotensin-aldosterone system

The RAAS is a complex system involved in the regulation of BP through water and electrolyte balance, and the preservation of vascular tone. The vasoconstrictive properties of RAAS include the activation of angiotensin (ANG) II, a potent vasoconstrictor also involved in cell proliferation, hypertrophy, generation of oxidative radicals and inflammation. ANG II also stimulates the secretion of aldosterone increasing the reabsorption of sodium into the blood, contributing to increases in BP.

Data from clinical investigations, epidemiological surveys and experimental studies suggest that the components of the circulating, as well as tissue-based RAAS, are markedly affected by sex. In particular, men were reported to have higher renin activity than women regardless of age and ethnicity [47]. More recently, further important sex differences in elements of RAAS were reported showing that older men have lower aminopeptidase A and angiotensin-converting enzyme (ACE) serum activity compared with older women, while older women have higher ACE2 serum activity than younger women [48]. During sodium intake, compared with women, men were also found to have significantly higher plasma aldosterone levels, extracellular volume and systolic BP, as well as lower adrenal response to exogenous ANG II [49].

These sex differences could be attributed to differences in the balance of the RAAS between the sexes, with the so-called depressor arm of the RAAS, i.e., ACE2/ANG 1-7/Mas receptor and ANG type 2 receptor (AT2R) counter-regulating the classical pressor ACE/ANG II/AT1R pathway [50]. In this context, it was suggested that men have the pressor pathway enhanced, while premenopausal women have mainly the depressor pathway activated, whose activity is decreased in older women [51].

Experimental animals also demonstrate significant sex differences. In particular, young male SHR have higher mean BP than young female SHR [52–54], while this sex difference is eliminated by RAAS inhibition [55]. This also occurs after cessation of estrous cycling, which is due to an increase in BP in females rather than any change in BP in males [56, 57]. The sex-specific regulation of the RAAS includes higher renal angiotensinogen mRNA and protein levels in old males than females, as well as higher renal ANG II in old females than males, suggesting sex differences in the depressor response to AT1R even when BP is similar [58]. In addition, endothelin (ET) was shown to mediate increases in BP in old female SHR [59]. Taken together, these data highlight the important role of the RAAS and ET mediating the increase in BP subsequent to cessation of estrous cycling in aging female SHR.

In line with these findings, it has been widely hypothesized that E2 is a protective CV factor due to its capacity to control various components of the RAAS (Fig. 2b). For example, HT was associated with reduced renin levels and increased angiotensinogen levels [16], as well as reduced circulating levels of ACE activity [17], which may be mediated in part by a direct down-regulating effect of E2 on ACE mRNA [60]. Treatment of OVX rats with E2 led to reduced tissue responsiveness to ANG II and attenuated ANG-induced aldosterone secretion [61, 62], as well as reduced AT1R levels in ANG II-target tissues [62–64]. In addition, the administration of E2 significantly increased the production of ANG 1-7 [65, 66], an angiotensin with cardioprotective effects. Overall, E2 appears to attenuate the production of ANG II and the levels of AT1R, thereby leading to a decreased RAAS activity.

### Body mass

Higher body mass is associated with increased risk for the development of HTN [67, 68], underlain by increased sympathetic activity, increased ANG II formation and renin release, leading to adrenal aldosterone production, thereby resulting in sodium retention. Increased visceral fat is associated with increased inflammatory mediators, increased oxidative stress and decreased endothelial vasodilation [8, 69].

Not surprisingly, one sex may be more vulnerable than the other. In particular, women may have higher levels of body fat (adipose tissue) compared with men and a greater risk of developing metabolic syndrome [70]. Notably, overweight and obese women have a higher risk of developing HTN compared with overweight and obese men [71]. Decreased E2 levels after menopause are markedly associated with lipid profile variations and abdominal fat accumulation. Therefore, alterations in E2 levels may lead to metabolic and adipocyte physiology disturbances contributing to obesity (Fig. 2c).

Subcutaneous and visceral adipose tissues express both ER, ERα, and ERβ, with ERα playing a major role in the activity of adipocytes and sex-specific fat distribution [72, 73]. It has been shown that non-classical ERα signaling mediates major effects of E2 on energy balance, suggesting that selective ERα agonists could reduce the risk of obesity and metabolic alterations in postmenopausal women [74]. In addition, E2 directly increased the number of anti-lipolytic α2A-adrenergic receptors in subcutaneous adipocytes in vivo and in vitro [75], but not in intra-abdominal adipose tissue, and it increased the lipolytic β-adrenergic receptor expression through ERα in vitro [76]. Further complex regulatory effects of E2 include the down-regulation of peroxisome proliferator-activated receptor-γ in the adipose tissue and concomitant adipogenesis-related genes [77], as well as the up-regulation of peroxisome proliferator-activated receptor-α in the liver [78]. These results provide a mechanistic insight for the effects of E2 on the maintenance of fat distribution with an increased use of lipids as an energy source, which may partially promote the fat reduction in abdominal fat [79].

The regulation of adipokines and cytokines released by the adipose tissue can be impaired in obesity and metabolic syndrome, thereby contributing to CV complications, including arterial stiffness, vascular and renal damage, ultimately contributing to the development of HTN [6, 80]. Adipokines, such as leptin (a metabolic regulator and feedback signal of body fat to regulate appetite also with a lipolytic effect) and adiponectin (an anti-inflammatory hormone) are cytokines released by the adipose tissue. These hormones have gained attention due to their capacity to influence the inflammatory system with pro- and anti-inflammatory actions. Increased levels of leptin and resistin—another adipokine that contributes to obesity—and lower levels of adiponectin were previously associated with uncontrolled BP [80]. In obese postmenopausal women, increased leptin and resistin levels and decreased adiponectin levels were reported, while HT was shown to be beneficial in reducing many of the parameters of metabolic syndrome [81]. In addition, E2 may increase leptin sensitivity by controlling the expression of leptin-specific receptors [24]. Furthermore, the administration of estradiol benzoate reduced resistin levels in adipocytes [82]. Together, these data suggest that E2 is a pivotal regulator of adipokines.

### Oxidative stress

Oxidative stress is a condition that occurs when the rate of reactive oxygen species (ROS) formation exceeds the rate of the antioxidant defense system. ROS has an important role in cell signaling and tissue homeostasis. In pathological conditions and environmental stress, ROS levels can increase dramatically and may result in significant damage to cellular structures. Oxidative stress has been linked to the development of HTN.

Women prior to menopause appear to have lower levels of oxidative stress compared with age-matched men, mediated by anti-oxidant activities of E2 scavenging-free radicals [83–85]. In contrast, postmenopausal women have higher oxidative stress levels than age-matched men [86]. Experimental data suggest a greater antioxidant potential in females over males [87], with higher levels of superoxide generation and lower levels of nitric oxide (NO) in males compared with females [88, 89]. Furthermore, female SHR showed increased renal NADPH oxidase activity and urinary F2-isoprostanes compared with male SHR [88, 90], corroborating the idea that E2 has a prominent role in sex differences in oxidative stress (Fig. 2d).

In OVX rats, E2 deficiency was associated with increased H_2_O_2_ production, as well as increased AT1R levels, leading to increased oxidative stress and endothelial dysfunction [64, 91]. In contrast, the administration of E2 in OVX rats led to the down-regulation of AT1R and E2 treatment of vascular smooth muscle cells resulted in a time-dependent downregulation of AT1R mRNA [64], indicating that the antioxidant properties of E2 may be mediated through the downregulation of the AT1R gene expression. In addition, E2 stimulates NO level increases in endothelial cells via endothelial NO synthase activation and ER-mediated mechanisms [18, 92, 93]. Ovariectomy-related loss of E2 leads to reduced endothelial NO synthase levels and activity [94], which appears to exert broad cardioprotective actions, including the attenuation of chemotherapeutic drug-induced cardiomyopathy [95]. E2 was further shown to reduce NADPH oxidase levels in endothelial cells [96], as well as to increase the levels of the antioxidant enzyme heme oxygenase 1 in in vivo and in vitro models [97–102]. Also, E2 administration in OVX rats led to an increase in catalase activity [103–105], and acute E2 treatment substantially enhanced myocardial catalase activity and restored LV oxidative stress and dysfunction caused by ethanol in OVX rats [106]. Overall, E2 modulates several factors, including pro- and antioxidant enzymes, thereby attenuating the production of ROS.

### Inflammation

Inflammation plays a central role in the CV system, underlying several CV pathologies. CV alterations trigger activation of inflammatory responses due to systemic damage, releasing pro- and anti-inflammatory factors, and activating cellular stress pathways.

Adipose tissue dysregulation is one of the main sources of inflammatory signaling in obesity-associated metabolic alterations, and E2 plays an important role. Menopause is associated with increases in fat mass, as well as elevated circulating inflammatory markers, such as tumor necrosis factor alpha (TNFα), interleukin-6 (IL-6) and plasminogen activator inhibitor-1 [107, 108]. Similarly, the loss of E2 in OVX rodents, as well as the deficiency of ERα, has been linked with increased adiposity in part mediated by increased food intake and decreased energy expenditure, accompanied by increased inflammation, while E2 treatment attenuated these effects [109–111]. In fact, ERα protects against obesity-related diseases and inflammation [112, 113]. In this context, ERα polymorphisms result in insulin resistance, body fat accumulation and inflammation [114, 115].

Many of the alterations occurring in comorbidities associated with decreased E2 levels and decreased ER expression, including the metabolic syndrome and obesity accompanied by inflammation, are attenuated by E2 (Fig. 2e). In particular, the administration of E2 has been shown to be important in the regulation of metabolic and inflammatory processes, leading to decreased expression of genes involved in lipogenesis [116], increased glucose clearance [117], lowered levels of inflammation soluble intracellular adhesion molecule 1, vascular cell adhesion molecule 1, E-selectin and ET in postmenopausal women [19, 118], as well as lowered circulatory cytokine levels, including TNFα, IL-1β and IL-10, in OVX rats [119]. In addition, E2 induces the transcriptional activation of Fas ligand via an ER-mediated, NO-dependent mechanism, thereby resulting in the inhibition of leukocyte traffic across the endothelium [120].

E2 also has a key role in the regulation of NFκB, which is a central modulator of a variety of inflammatory pathways and cellular responses. In particular, E2 repressed the activity of NFκB by inhibiting its DNA-binding ability, as well as reducing the NFκB-p65 subunit expression, thereby down-regulating NFκB-dependent activation of genes, such as TNFα and IL-6 [121, 122]. In addition, E2 appears to suppress inflammatory cell adhesion to endothelial cells via an ERα-dependent mechanism, which may involve inhibition of NFκB-mediated up-regulation of vascular cell adhesion molecule 1 [123]. Overall, E2 is important in the regulation of inflammatory pathways and signaling.

### Endothelial dysfunction

Endothelial dysfunction is associated with increased systemic oxidative stress and vascular inflammation. It is characterized by reduced levels of vasodilators, such as NO, and increased ET levels, thereby modulating vascular tone.

Clinical data have indicated an important association between endothelial dysfunction and reduced E2 levels in postmenopausal women. In particular, vascular and hemodynamic parameters and arterial stiffness were elevated, while the endothelial function was reduced across different stages of the menopausal transition [124]. In line, oophorectomy associated with acute E2 deprivation resulted in impaired endothelium-dependent vasodilation, due to reduced NO availability [125], while HT improved endothelium-dependent vasodilation after oophorectomy, as well as after menopause [126]. Interestingly, this beneficial effect of HT was reported to be greater in hypertensive postmenopausal women [127]. Furthermore, the levels of plasma ET were higher in postmenopausal women than in premenopausal women, while HT attenuated this increase [128].

E2 may act on the CV system directly in the vessels or indirectly by regulating the lipid profile (Fig. 2f). These E2 actions are associated with lower coronary vascular resistance, enhanced coronary blood flow and improved coronary vasodilatory responses [129, 130]. The effects of E2 in the vascular system comprise increases in the synthesis of NO [92], modulation of serum- and ANG II-stimulated synthesis of ET [131, 132], and long-term modulation of vascular tonus, inducing the production of prostaglandins [133]. Along this line, E2 administration in OVX rats restored flow-induced dilation mediated by epoxyeicosatrienoic acids, which are generated following metabolism of arachidonic acid by cytochrome P450 epoxygenases [134–136]. However, it is not clear whether E2 regulates the synthesis of epoxyeicosatrienoic acids. E2 also has an anti-proliferative role in vascular remodeling [137], inhibiting the proliferation of the inner layer after injury [138]. Studies have also demonstrated antioxidant effects of E2. In particular, E2 appears to be involved in the regulation of the uptake of oxidized low-density lipoprotein, which was found to be dependent upon ER activation [139]. In addition, E2 reduced cholesteryl ester accumulation in human monocyte-derived macrophages [139]. Collectively, these data indicate that E2 regulates endothelial function through multiple mechanisms.

### Salt sensitivity

Salt sensitivity refers to BP responses to changes in dietary salt intake. It has been described that salt intake has pathological effects on the vasculature and sodium homeostasis, and salt sensitivity appears to be related not only to kidney malfunction but also to endothelial dysfunction [140]. Interventional studies with essential hypertensive patients receiving diets with varying salt levels demonstrated that patients who were salt-sensitive more often had LVH, CV events, and/or endothelial dysfunction than non-salt sensitive hypertensive patients [141–143].

A recent study with human subjects and experimental animals indicated that females have 30% higher salt sensitivity of BP than males, regardless of menopausal status or HTN and altered aldosterone production, and differences in the kidney seem to be responsible for this sex-specific effect [144]. Another previous study examined BP responses to dietary sodium and potassium interventions by sex, age and baseline BP subgroups among men and women aged 16 years or older. Also, this study showed that the female sex, as well as older age and elevated baseline BP levels, increases BP responses to dietary sodium intake [145]. Salt sensitivity increases with age and is likely mediated by impaired vasodilation of the renal circulation, possibly due to reduced NO availability, increased vasoconstriction response to ANG II, leading to a disturbed renal sodium handling, oxidative stress, and HTN [146, 147]. As postmenopausal women appear to be more salt-sensitive than pre-menopausal women [148], decreases in ovarian hormone levels and increased sensitivity to dietary sodium may be important factors in the development of HTN at menopause. Furthermore, the surgical removal of the ovaries is associated with the development of salt sensitivity [149], while the administration of E2 reduced salt sensitivity of BP in postmenopausal women [150]. These data support further the view that salt sensitivity may be associated at least in part with changes of the hormonal profile, particularly E2 (Fig. 2g), that occur in women after menopause.

HTN due to salt sensitivity has been linked to decreased renal NO production and inappropriate activation of the RAAS [151]. As previously discussed, E2 has an important role in both systems, NO and RAAS, and through its antioxidant properties, it has the ability to increase the bioavailability of endothelium-derived NO. Subsequently, in the presence of salt sensitivity, decreases in E2 levels may impair the bioavailability of NO. In addition, in OVX salt-sensitive rats, HTN was correlated with increased renal AT1R protein levels, while treatment with E2 or an AT1R antagonist prevented the development of HTN [151]. Therefore, menopause-related E2 deficiency leads to the over-expression of renal AT1R, thereby resulting in oxidative stress and disturbed renal sodium handling, ultimately contributing to the development of HTN.

### Genetic factors

It is well known that genetic factors play a major role in CV pathology [152, 153] and that they influence the development of HTN [154]. Interestingly, through modeling gene-environment interactions, several genetic variants associated with HTN-related phenotypes have been discovered [155]. Thus, mechanisms related to individual genetic variation may lead to specific responses in HTN, which may differ significantly between the sexes [156].

In this context, various studies have reported sex-specific associations between HTN and polymorphisms of components of the RAAS [157], endothelial NO synthase [158] and aldosterone synthase [159]. In a large-scale study of the general population, double homozygosity for Thr235 and Thr174 in the angiotensinogen gene was associated with a 10% increase in angiotensinogen levels and was considered a risk factor for elevated BP in women but not in men [157]. Gene variants of the endothelial NO synthase were reported to influence the long-term burden and trend of BP since childhood in females contributing to their predisposition to HTN [158]. Polymorphisms in the β_1_-adrenergic receptor and α_2A_ adrenergic receptor were also associated with BP in women [160]. Investigation of the association between the insertion/deletion (I/D) polymorphism of the gene that codes for ACE and HTN in black and white men and women revealed a significant association between the D variant and HTN only in black women, highlighting the importance of sex-specific ethnic differences in the association between genetic variation and expression of a hypertensive phenotype [161]. Furthermore, this *ACE* polymorphism was associated with BP in a sex- and age-dependent manner [162].

As previously mentioned, the effects of E2 in the CV system are mainly mediated by the two ERs, i.e., ERα encoded by the *ESR1* gene and ERβ encoded by the *ESR2* gene. Polymorphisms in *ESR1* have been associated with diastolic BP in women [163]. Moreover, *ESR1* genotypes and alterations in its expression have been linked with increased body mass and body fat distribution [115, 164, 165]. The binding of E2 to ERβ has been reported to lead to vasodilation [166]. In this context, it has been shown that women that are heterozygous for certain genotypic polymorphisms of *ESR2* present increased risk of HTN, especially those who use oral contraceptives [167], suggesting that specific single-nucleotide polymorphisms in *ESR2* may transform the interaction of E2 with ERβ to a harmful axis regarding BP instead of a protective one.

In addition, variation at rs10144225 in *ESR2* was associated with salt sensitivity of BP in premenopausal women but not in men or postmenopausal women [168]. In premenopausal women with the major allele, E2 is expected to bind to ERβ leading to vasodilation, thereby acting to protect against salt sensitivity of BP. However, in women with the risk allele, the binding affinity between E2 and ERβ may decrease, thereby attenuating the vasodilatory effects of E2, ultimately leading to salt sensitivity of BP. Together, polymorphic variants within *ESR2* may inhibit its binding to E2, thereby hindering the vasodilatory effects of E2, ultimately leading to a loss of its protective actions against HTN [168]. Interestingly, polymorphisms in the human follicle-stimulating hormone receptor gene, which may cause hereditary hypergonadotropic ovarian failure, have also been linked to HTN in women [169]. Therefore, genetic variation influences the regulatory effects of E2, thereby impacting sex-specific phenotypes in the development of HTN (Fig. 2h).

## Regulatory effects of estrogen in hypertension-induced target organ damage

Patients with HTN and lack of BP control have a higher probability to develop TOD, such as cardiac hypertrophy, vascular alterations—including arterial stiffness—and renal damage. Individuals can respond differently to the development and manifestation of the disease, response to treatment, outcome and recovery process. The development of these CV complications also differs significantly between men and women [156, 170, 171], and E2 appears to be crucial in sex-specific pathophysiology [172].

### Cardiac hypertrophy

The heart responds to pathological stimuli, such as HTN, aortic stenosis, or cardiac injury, with hypertrophy of the cardiac muscle, accompanied by several tissue and cellular alterations, including increases in cardiomyocyte size and changes in the extracellular matrix. Although initially this is an adaptive and compensatory response, upon the persistence of the stress factor, there is maladaptive remodeling leading to pathological hypertrophy. Consequently, HTN-induced LVH is a major risk factor for heart failure and sudden death [173]. In the hypertrophic process, distinct molecular mechanisms may occur between men and women, many of which are expected to be mediated by E2 (Fig. 2i).

Despite antihypertensive therapy, hypertensive women have a greater risk to develop LVH than hypertensive men [174], and the presence of LVH in HTN offsets the protection in a cardiovascular risk linked with the female sex [175]. As in patients with aortic stenosis [176–182], another major precursor inducing LVH, the hypertrophic response of the heart in hypertensive patients exhibits significant sex differences in its structural and functional adaptation [183, 184]. These differences include greater indexed LV mass, better systolic function and increased risk of incident heart failure with preserved ejection fraction in hypertensive women compared with hypertensive men [79, 185, 186]. Studies with experimental animals also demonstrate major sex differences in the development of HTN-induced LVH, where males develop greater hypertrophy and dysfunction than females [172].

Notably, HT in hypertensive postmenopausal women contributed to a reduction in LV mass [20, 187, 188], thereby indicating a modulatory role of E2 in HTN-induced LVH. Similarly, in OVX spontaneously hypertensive heart failure rats treated with E2, the development of HTN and related LVH were attenuated [189]. Direct effects of E2 and its receptors in the myocardium have been previously shown [190–198], which might affect several processes in a sex-specific manner [199–206]. The loss of E2 by ovariectomy suggests that E2 influences cardiac hypertrophy in part via the phosphoinositide 3-kinase (PI3K)/protein kinase B (PKB, also known as AKT) signaling pathway [94]. In this context, the hearts of premenopausal women exhibit greater PKB activity than those of men or of postmenopausal women, and treatment of rat cardiomyocytes with E2 led to higher levels of phosphorylated PKB [22]. Therefore, E2 influences signal transduction in the myocardium that might exert regulatory actions on the hypertrophic response in a sex-specific manner.

In addition, E2 inhibits the cardiomyocyte response to hypertrophic stimuli by preventing new protein synthesis and skeletal muscle actin expression [21]. ET stimulates the tyrosine phosphatase calcineurin resulting in new protein synthesis. Both ET and calcineurin were inhibited by E2, which also induced the gene encoding MCIP1, an anti-hypertrophic protein that prevents calcineurin activity [21]. E2 also stimulates the production and release of natriuretic peptides [21, 207], thereby inhibiting the hypertrophic response [208, 209]. Several studies revealed that E2 acts through ERβ to mitigate the deleterious signaling by ANG II that produces cardiac hypertrophy [208], as well as to protect against LVH in rodents with transverse aortic constriction [190, 210] in a sex-specific manner [201, 204, 211]. Interestingly, polymorphisms in the *ESR2* gene are associated with LV mass and wall thickness in women with HTN but not in men [212], thereby indicating an important role of ERβ in the development of cardiac hypertrophy and sex-specific responses.

### Arterial stiffness

Arterial stiffness is characterized by the reduced capability of an artery to expand and contract in response to pressure changes. This process is tightly associated with HTN, and arterial stiffness has emerged as an important predictor of CV events and mortality [213]. Sex differences in arterial stiffness have been reported, and E2 has been implicated in vascular and endothelial protection (Fig. 2j).

The structure of the arterial wall is maintained by the balance between collagen and elastin—extracellular matrix components responsible for the compliance and stability of the arterial wall. On the one hand, increased collagen content and density have been associated with increased vascular stiffness [214–217]. On the other hand, elastin is an essential determinant of arterial morphogenesis and vascular disease [218, 219]. In fact, mutations in the gene coding for the most abundant elastic fiber proteins result in a broad spectrum of elastic tissue disorders, ranging from skeletal abnormalities to ocular and vascular defects [220–226]. Along this line, elastin haploinsufficiency in mice leads to altered mechanical properties of large arteries, thereby contributing to increases in BP [227, 228]. Similarly, age-related proteolytic degradation and chemical alterations of elastic fibers result in changes in their mechanical properties [229], thereby conferring the arterial wall a more rigid structure, ultimately contributing to arterial stiffness.

Major determinants of arterial stiffness include age, sex and BP [230–233]. Accordingly, markers of arterial stiffness differ significantly between men and women. In particular, compared with elderly hypertensive men, elderly hypertensive women have a longer ejection time, earlier arterial wave reflection and smaller vessel size, independent of body size and heart rate [234]. Also in end-stage renal disease, arterial wave reflection is greater in women compared with men [235]. Furthermore, aortic stiffness is greater in women than men and is associated with diastolic dysfunction, impaired ventricular coupling and LV remodeling, potentially contributing to the greater risk of heart failure with preserved ejection fraction in women [236, 237]. Notably, compared with premenopausal women, postmenopausal women have greater pulse wave velocity, indicating that the deficiency of E2 associated with menopause may account for the augmented increase in arterial stiffness with aging in women [238–241]. Along this line, investigations of postmenopausal women indicate that HT ameliorates arterial stiffness [242, 243].

The molecular processes that contribute to the changes in vascular properties accounting for these differences are incompletely understood, as well as the role of biological sex influencing genes and proteins of the extracellular matrix in older males and females. Indeed, potential mechanisms include sex differences in collagen isoforms, elastin levels and abundance of other extracellular matrix proteins [244–246]. Along this line, the decrease of E2 at menopause may lead to arterial stiffness through mechanisms related to changes in the components of the arterial wall, such as collagen and elastin deposition, leading to alterations in the arterial biomechanical properties. In this context, E2 has been shown to decrease collagen deposition and increase elastin production in human aortic smooth muscle cells [23]. Notably, acute endogenous E2 deprivation leads to impaired NO release [125], thereby resulting in loss of vasodilation, ultimately contributing to arterial stiffness. However, E2 administration promotes vasodilation in part by stimulating endothelial NO synthase and NO release, thereby promoting vasodilation [247, 248], as well as by up-regulating the endothelial NO synthase messenger RNA and protein levels [18, 93]. E2 also inhibits vascular smooth muscle cell proliferation [249] mediated by ERα [138, 250]. Therefore, E2 exerting direct effects in the vessel wall contributes to sex differences in arterial stiffening.

### Renal dysfunction

Chronic kidney disease (CKD), defined by albuminuria and/or reduced estimated glomerular filtration rate, is a common TOD in HTN, associated with high rates of morbidity and mortality [251]. The prevalence of CKD increases with aging, underlain by changes in kidney morphology, hemodynamics and function, which increase the incidence of CV events. Sex differences in CKD have been reported, and E2 has been shown to influence renal disease development (Fig. 2k).

Considering chronic renal disease of various etiologies reveals a complicated role for biological sex. For example, the prevalence of CKD appears to be higher in older women than older men [252, 253]. However, the male sex is an independent risk factor for end-stage renal disease [254]. Further studies have reported different clinical features and prognosis of renal diseases between men and women. In particular, men present with a more rapid rate of progression of renal diseases, such as polycystic kidney disease, IgA nephropathy, membranous nephropathy, chronic renal failure, than women [255, 256]. Similarly, there is a faster decline in renal function and worse prognosis of CKD in men than in women [257, 258]. Collectively, these data indicate that the male sex is a major determinant of the progression of renal dysfunction, while younger, premenopausal women may be protected from the development of CKD.

Experimental studies of CKD with rats also showed that males present with faster progression and worse outcome of renal disease than females [259]. In particular, male rats exhibit marked albuminuria, augmented cortical histological damage, interstitial inflammation and fibrosis, while these are all significantly less pronounced in female rats [260]. Similarly, renal function is worse in male than in female rats following ischemia/reperfusion injury [261]. However, a more recent study reported no sex differences in acute renal injury due to ischemia alone, but only male rats developed CKD [262]. Notably, OVX rats also developed CKD and ovariectomy was associated with increased proteinuria, oxidative stress, increased glomerular and tubular damage, whereas E2 is thought to protect against renal disease [25, 262, 263]. Along this line, HT has been suggested for the management of CKD in postmenopausal women [264].

Although the molecular processes regulated by E2 that might affect renal function are incompletely understood, E2 exerts modulatory actions on renal morphology, such as anti-growth effects on glomerular mesangial cells and inhibition of mesangial extracellular matrix accumulation, common to the development of glomerular sclerosis [265–268]. In particular, E2 inhibits collagen synthesis induced by transforming growth factor β in glomerular mesangial cells, suggesting that E2 may limit the progression of glomerulosclerosis, thereby attenuating deleterious effects in the kidney [266, 268, 269]. In addition, the inhibitory effects of E2 on various components of the RAAS may protect the kidney against glomerular remodeling, damage and glomerulosclerosis. On the other hand, the stimulatory effects of E2 on NO may attenuate mesangial cell growth, matrix production, vasoconstriction and renal sodium reabsorption, which contribute to the progression of CKD [270]. It has been suggested that the relative renal protection observed in females may be mediated by ERα [271]. Further research is warranted.

## Perspectives and significance

Although the role of biological sex has yet underestimated consequences for physiology and pathology [272], several experimental and clinical studies have demonstrated the importance of understanding its effects and the underlying mechanisms in many diseases, highlighting that sex differences represent important biological phenomena that need further investigation. At least in part, E2 accounts for these sex differences and has a key role in the development of HTN and associated TOD. However, there are several pitfalls of HT and risks that depend on the type, dose, duration of use, route of administration and timing of initiation [273]. In this context, the elucidation of E2-related mechanisms and the identification of targets with therapeutic potential will contribute to the development of more efficient therapies for men and women, improving the treatment and care of patients with HTN and CV diseases according to individual needs.

## Data Availability

Not applicable.

## Abbreviations

ACE: Angiotensin-converting enzyme
ANG: Angiotensin
AT1R: Angiotensin type 1 receptor
BP: Blood pressure
CV: Cardiovascular
E2: 17β-Estradiol
ER: Estrogen receptor
ET: Endothelin
HT: Hormone therapy
HTN: Hypertension
IL-6: Interleukin-6
LV: Left ventricular
LVH: LV hypertrophy
NO: Nitric oxide
OVX: Ovariectomized
RAAS: Renin-angiotensin-aldosterone system
ROS: Reactive oxygen species
SHR: Spontaneously hypertensive rats
SNS: Sympathetic nervous system
TOD: Target organ damage
